# Assessing Population Aging and Disability in Sub-Saharan Africa: Lessons from Malawi?

**DOI:** 10.1371/journal.pmed.1001441

**Published:** 2013-05-07

**Authors:** Andreas E. Stuck, Lyson Tenthani, Matthias Egger

**Affiliations:** 1 Geriatrics Department, University of Bern, Inselspital University Hospital, Bern, Switzerland; 2 Department of HIV and AIDS, Ministry of Health, Lilongwe, Malawi; 3 Institute of Social and Preventive Medicine (ISPM), University of Bern, Switzerland; 4 School of Public Health & Family Medicine, University of Cape Town, Cape Town, South Africa

## Abstract

Andreas Stuck and colleagues discuss research by Collin Payne and colleagues on disability in older Malawi adults and next steps for research and policy.

*Please see later in the article for the Editors' Summary*

**Linked Research Article**

This Perspective discusses the following new study published in *PLOS Medicine*:

Payne CF, Mkandawire J, Kohler H-P (2013) Disability Transitions and Health Expectancies among Adults 45 Years and Older in Malawi: A Cohort-Based Model. PLoS Med 10(5): e1001439. doi:10.1371/journal.pmed.1001435

Collin Payne and colleagues investigated development of disabilities and years expected to live with disabilities in participants 45 years and older participating in the Malawi Longitudinal Survey of Families and Health.

In this week's issue of *PLOS Medicine*, Collin Payne and colleagues report a study of the burden of physical limitations and disability in rural Malawi, with a focus on disability transitions and health expectancies for adults aged 45 years and older, the age at which individuals in this low-income country begin to experience significant disability [1]. The study is based on the Malawi Longitudinal Survey of Families and Health (MLSFH), which collected data on functional limitations, socioeconomic conditions, biomarkers, and health expectations, and performed HIV testing and counseling. The authors used the longitudinal data to estimate transition probabilities between disability states and microsimulation to calculate health expectancies for years of healthy, moderately limited, and severely limited life.

## A Landmark Study for Malawi

The study of Payne and colleagues is, to our knowledge, the first empirical study to report disability states, and to estimate transitions between them, for Malawi's population of 45 years of age and older. The study provides detailed estimates for healthy life expectancy (HALE, an estimate of equivalent years of good health), which differ from those recently published by the Global Burden of Disease study (GBD) [2],[3]. The GBD estimates that in Malawi 50-year old women can expect to live 76.1% of their remaining 23.4 years in good health and 50-year old men can expect to live 76.7% of their remaining 20.6 years in good health [2]. In contrast, Payne and colleagues estimated that women aged 45 years spend only 42% of 28.0 remaining years in good health, and men 59% of 25.4 years.

These differences most likely are explained by different methodological approaches. The GBD study is based on epidemiological information on morbidity, mortality, and risk factors [2],[3]. The loss of life years due to disability is indirectly measured by attaching a disability weight to each type of disease or condition. In contrast, the estimates from the MLSFH were based on self-reported disability, and healthy life expectancy was calculated by subtracting years spent with limitations.

## Strengths and Weaknesses

Payne and colleagues' study was based on state-of-the-art transitional analyses of a longitudinal dataset and provides easy-to-understand information on remaining life expectancies with and without disability. One limitation is that it is based on data that are unlikely to be representative for rural Malawi. The MLSFH included selected villages from three out of 28 districts. The Third Integrated Household Survey has shown relevant differences in chronic care burden between these districts [4]. Also, HIV prevalence was surprisingly low in this sample (3%). National reports estimate HIV prevalence at about 15% among 45- to 50-year old persons, but no data are available on older people [5]. As described in detail in the supporting information (specifically, Text S1 and Table S8) of their paper, the sampling strategy of the MLSFH was complex, differed between sites, and was not designed to result in a representative sample; and attrition was substantial (25.6% in 2010, related to migration, mortality, and other factors) [1].

An important strength is the assessment of functional disabilities using questions on activities such as tending to cattle, carrying heavy loads, pounding maize, or digging a pit latrine. These measures adequately reflect the situation in rural Malawi, but environmental demands will be somewhat different for the 15% of the population living in urban settings. Also, some activities may be more relevant to daily living than others. For example, digging a pit latrine is a physically very demanding task, which a male family member typically has to perform only about once per year. Also, the relevance of activities may change in the future. For example, increasingly diesel-engine-driven grain mills are available so that the strenuous task of pounding maize might become less relevant.

## Need for Further Research

Payne and colleagues found that functional limitations might often be reversible: as many as 41% of persons with mild functional limitation recovered to a healthy state without functional limitation. An in-depth understanding of the factors associated with transitioning back to a healthy state is needed to develop interventions. For example, about half of moderately and the majority of severely limited individuals stated that pain interfered with their normal work during the past 4 weeks, suggesting that identifying causes of and effective treatments for the pain experienced could improve an individual's ability to function.

Risk factors and causes of disability are key to developing preventive and rehabilitative interventions and policy making and should be addressed in future surveys. Based on the GBD 2010 estimates, the modifiable risk factors in Eastern sub-Saharan Africa that are most important in terms of attributable burden of disease in older men and women include high blood pressure, smoking, alcohol use, dietary risk factors, and household air pollution [6]. In the age group 60–64 years, important causes of disability include cardiovascular and circulatory diseases (24% of disability-adjusted life years [DALYs]), HIV/AIDS and tuberculosis (10%), pneumonia and other infections (10%), and cancer (7.5%) [3]. With continuing and increasing access to antiretroviral therapy, HIV prevalence rates will likely increase in older cohorts in Malawi and elsewhere, and add importantly to the chronic disease burden of disability among elderly individuals [7]. Finally, health policy will need information from subregional studies [8]. National and regional studies of the burden of disease are important but the high level of resolution will often hide substantial heterogeneity in health, and the relative importance of different causes and risk factors across federal states, provinces, or other subdivisions, as recently demonstrated for Mexico [9].

## Aging and Disability: A Priority for Health Policy

The higher estimates of burden of disability in Malawi defined by Payne and colleagues compared with previous estimates must be of concern, and the same may be the case for other low-income countries. These high rates of disability result in major economic loss, with an expected increase in losses in the future due to demographic changes. Disability affects both paid and unpaid work. For example, many older people are needed as carers, such as in the context of children orphaned by HIV/AIDS. In Malawi, 19% of children are not living with a biological parent, with many under the care of older members of the extended family [5]. Furthermore, individuals with functional limitations might need help with activities of daily living, resulting in a burden for formal and informal support systems.

This study emphasizes that health policy should urgently address disability in older persons, an issue largely ignored at present. This includes primary prevention aimed at the population at large and targeted interventions in old age. Population prevention strategies should focus on lowering blood pressure, reducing smoking and alcohol use, and promoting efficient stove and fuel technologies to combat household air pollution and deforestation. Many older persons will be affected by multiple risks and morbidities, which will require the careful evaluation of affordable and effective multimodal interventions ranging from prevention to rehabilitation, such as implementing a health risk appraisal followed by tailored interventions [10], offering a polypill approach [11], or addressing uncontrolled pain. Investments in improving health in older people will not only improve quality of life across generations, but bring economic return for generations to come.

## Abbreviations

DALY: Disability-Adjusted Life Year
GBD: Global Burden of Disease study
HALE: Health-Adjusted Life Expectancy
MLSFH: Malawi Longitudinal Survey of Families and Health
